# Dermatological manifestations relating to nutritional deficiencies after bariatric surgery: case report and integrative literature review

**DOI:** 10.1590/1516-3180.2021.0616.R1.17022022

**Published:** 2022-08-29

**Authors:** Andressa Christine Ferreira Silva, Laura Moya Kazmarek, Elemir Macedo de Souza, Maria Letícia Cintra, Fernanda Teixeira

**Affiliations:** I MD. Physician, Department of Pathology, Faculdade de Ciências Médicas da Universidade Estadual de Campinas (FCM-UNICAMP), Campinas (SP), Brazil.; II MD. Physician, Department of Pathology, Faculdade de Ciências Médicas da Universidade Estadual de Campinas (FCM-UNICAMP), Campinas (SP), Brazil.; III MD, PhD. Associate Professor with Tenure (Phased Retirement), Department of Dermatology, Faculdade de Ciências Médicas da Universidade Estadual de Campinas (FCM-UNICAMP), Campinas (SP), Brazil.; IV MD, PhD. Professor and Head, Department of Pathology, Faculdade de Ciências Médicas da Universidade Estadual de Campinas (FCM-UNICAMP), Campinas (SP), Brazil.; V MD, PhD. Consultant Professor, Department of Pathology, Faculdade de Ciências Médicas da Universidade Estadual de Campinas (FCM-UNICAMP), Campinas (SP), Brazil.

**Keywords:** Bariatric surgery, Skin manifestations, Malnutrition, Skin diseases, Deficiency diseases, Xerophthalmias, Phrynoderma, Acrodermatitis enteropathica, Bariatric surgical procedures, Nutritional deficiencies, Dermatopathies

## Abstract

**BACKGROUND:**

The number of bariatric surgeries performed worldwide is growing. Among the main short, medium or long-term complications after surgery are nutritional deficiencies. Many of these, such as those of Zn, Cu and vitamins A, B1, B3, B6 and B12, are manifested by dermatological lesions before potentially fatal systemic disorders occur.

**OBJECTIVE:**

To identify the main dermatological manifestations associated with nutritional deficiencies after bariatric surgery, and the associated variables.

**DESIGN AND SETTING:**

Integrative literature review carried out at a public university in Brazil.

**METHODS:**

This was a case report and a review of health research portals and databases of national and international biomedical journals, without publication date limitation. The descriptors used for searches followed the ideal methodology for each database/search portal: “bariatric surgery”, “skin”, “skin disease”, “skin manifestation”, “deficiency disease” and “malnutrition”.

**RESULTS:**

A total of 59 articles were selected, among which 23 were review articles or articles that addressed specific dermatological manifestations. The other 36 articles described 41 cases, which were organized into a table with the clinical variables.

**CONCLUSIONS:**

Although nutritional deficiencies are expected as complications after bariatric surgery, few articles relating them to their dermatological manifestations were found. It is important to recognize skin changes caused by nutritional deficiencies in patients treated via bariatric surgery, as these may occur before systemic complications appear and are easier to diagnose when the patient does not have any systemic symptoms yet. However, there is generally a delay between the appearance of skin lesions and making the diagnosis of nutritional deficiency.

## INTRODUCTION

Obesity is associated with decreased quality of life,^1^ comorbidities and increased mortality.^2^ Cancer is also associated with obesity, particularly in the breast, endometrium, colon and prostate.^3^ Obesity is probably an independent risk factor for greater severity of COVID-19^4^ and decreases protection in immunization against influenza.^5^

The growing incidence of obesity and overweight is among the biggest public health problems worldwide.^6^

As a result, the number of bariatric surgeries, which are considered to be an effective treatment for obesity (and diseases resulting from it),^7^ is growing.

Nonsurgical treatment modalities may promote some weight loss, but it is usually not maintained over the long term because patient compliance is generally not adequate. Surgical treatment is attractive for patients because it enables a more pronounced weight loss that can be sustained over the long term, in addition to having a positive impact on comorbidities associated with obesity, thereby reducing mortality and improving quality of life. However, like any treatment, bariatric surgery is not without complications, whether perioperative (such as bleeding, infection or thrombotic events) or malabsorptive (which would lead to or worsen vitamin and mineral deficiencies). The indications for surgery need to be individualized for each patient's condition; rigid criteria do not apply, and risk/benefit ratios should always be considered.^8^

There are three main categories of bariatric surgery:^9^ 1 – restrictive: gastric banding, sleeve gastrectomy, banded sleeve gastrectomy and intragastric balloon; 2 – predominantly restrictive: Roux-en-Y gastric bypass, with or without retention ring; and 3 – predominantly malabsorptive: biliopancreatic shunt with sleeve gastrectomy, with or without distal gastric preservation, and biliopancreatic shunt with sleeve gastrectomy and pyloric preservation. Roux-en-Y is the type of surgery most performed, followed by sleeve gastrectomy and gastric banding (these three procedures make up 92.2% of those performed worldwide).10 Among the main complications reported are nutritional deficiencies, which can be due to great weight loss, malabsorption of nutrients and/or changes to the patient's dietary habits.

The skin is one of the most effective health indicators. Changes in color and texture can be a sign of systemic dysfunctions.^11^ Knowing the dermatological changes relating to vitamin and mineral deficiencies that appear after bariatric surgery can help in their identification, thus preventing evolution of the patient to systemic disorders.

## CASE REPORT

What prompted us to start this study was witnessing the evolution of a teenager, a 17-year-old Caucasian female, who had undergone Roux-en-Y gastric bypass surgery at age 15 years. Before the surgery, she was a normal, albeit obese girl. After surgery, she received vitamin B12 and folic acid supplementation regularly.

**Figure 1:**
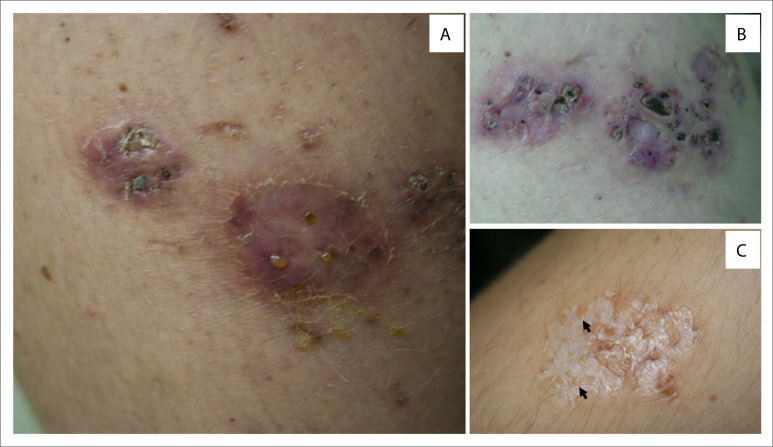
Acquired perforating dermatosis secondary to nutritional deficiency after bariatric surgery. A: Nodules of various sizes, erythematous and pigmented, with multiple sinuses exuding a viscous, yellow fluid. Some of the sinuses are closed by scabs. B: With treatment, the lesions became flat and the sinuses closed. Redness and swelling progressively resolved. C: Lesions resolved with white atrophy and sinus sites were replaced by anetodermal scars and residual pseudothesaurismotic papules (arrows).

At age 16 years, she complained of painful areas on her thighs and buttocks, 5 mm to 20 mm in diameter, which progressively became larger, more numerous, deeper and more painful, suggestive of erythema nodosum. Soon afterwards, the areas showed numerous sinuses that expelled a viscous yellow fluid (Figure 1A). She also complained of chronic diarrhea and decreased visual acuity.

On examination, she looked tired, pale and distressed. Her body mass index was 31 kg/m2. Her skin lesions were now quite numerous and measured up to 30 mm in diameter. A punch biopsy was obtained with the following differential diagnoses: acquired perforating dermatosis due to vitamin deficiencies, deep mycosis, atypical mycobacterial infection, pyoderma gangrenosum and subcutaneous Sweet's syndrome. Ophthalmological examination showed keratinization of the bulbar conjunctiva, suggestive of hypovitaminosis A.

**Figure 2:**
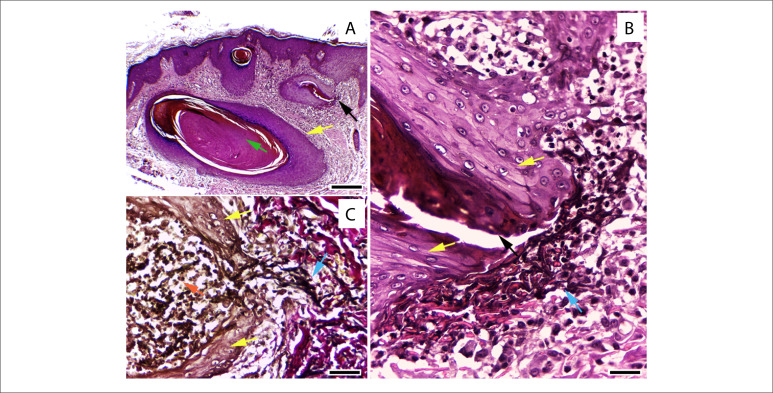
Acquired perforating dermatosis secondary to nutritional deficiency after bariatric surgery. A: horn (green arrow) cyst (yellow arrow) and open distorted follicle (black arrow); B: follicle with hyperplastic epithelium (yellow arrows), with perforation (black arrow), close to the degenerated elastic fibers (blue arrow); C: the same follicle (yellow arrows) stained using Verhoeff-van Gieson method, containing polymorphonuclear cells (red arrow), showing transepithelial elimination of black elastic fibers (blue arrow). Hematoxylin and eosin (H&E) x 100 (A) and x 400 (B). Verhoeff-van Gieson x 400 (C). Scale bar: 0.25 mm (A); 0.05 mm (B, C).

Blood tests results showed the following: vitamin A: 0.1 mcm/l (normal range 1.2–4.2); vitamin E: 3.7 μg/ml (normal range 15–40); Hb: 9.5 g/dl (normal range 12–15.5); iron: 43 mcg/dl (normal range 60–170); and ferritin: 7.2 ng/ml (normal range 10–120) The lipid profile was normal, except for the triglyceride levels, which were very low (27.2 mg/dl). Histological examination of the skin showed horn cysts, perforating folliculitis with transepithelial elimination of elastic fibers and fibrosis (Figure 2).

She was treated with supplementation of oral fat-soluble vitamins and B vitamins, iron and calcium. After thirteen months of this, all the skin lesions had resolved, albeit with considerable scarring (Figures 1B and 1C). Lifetime monitoring was instituted to prevent recurrent nutrient deficiency conditions.

This impressive case raised the issues of postoperative vitamin and mineral deficiencies and of patient compliance, which will be discussed in this paper. However, it was also a sad reminder of patients’ and their parents’ expectations and the ethics regarding bariatric surgery in the pediatric population; issues that will not be tackled here.^12^

## METHODS

A literature search was performed, without publication time restriction, up to 2020, through the following biomedical databases: PubMed/MEDLINE (www.ncbi.nlm.nih.gov/pubmed), Virtual Health Library (https://bvsalud.org/), SCOPUS (www.scopus. com), EMBASE (www.embase.com), Web of Science (www.webofknowledge.com) and SciELO (https://scielo.org/en/). The following descriptors were used: “bariatric surgery”, “skin”, “skin disease”, “skin manifestation”, “deficiency disease”, “malnutrition” and “undernutrition”; and, for EMBASE, equivalent descriptors.

The eligibility criteria were the following: (a) systematic reviews, meta-analyses, case reports and case series among post-bariatric patients of any sex and age who presented dermatological manifestations resulting from nutritional deficiencies; (b) the languages English, French, Italian, Portuguese and Spanish. The information contained in each text was synthetized and recorded on protocol sheets, so that the team members could verify their validity and applicability and, possibly, find elements to support the discussion that would lead to interpretation of the data. These data were manually collected and stored in spreadsheet format, using the Microsoft Excel 2013 software (Microsoft Windows, Redmond, WA, United States).

The studies that were retrieved through searches in the databases were compared individually. None of the participants in this review were blinded to the titles of journals, studies, authors or even the institutions to which the authors were affiliated. Duplicate or split studies were computed and analyzed as single studies, thereby avoiding repetition and data overlap. In the case of partially published studies or studies still under development, only the most recently published information was considered. Doubts about the suitability and eligibility of each study were discussed by the team. The main dermatological manifestations associated with nutritional deficiencies in patients treated by some type of bariatric surgery and the proposed etiopathogenesis of such processes were discussed.

Exploratory data analysis was performed using summary measurements (mean, standard deviation, minimum, median, maximum, frequency and percentage). To assess associations between the variables, a meta-analysis was performed on individual participant data (IPD meta-analysis) using logistic regression models with mixed effects, through the Laplace method. The significance level adopted was 5%.

The patient's mother was shown the pictures included in this article and consented, in writing, to their publication. This document is in our custody and can be produced for perusal. The institutional Ethics Committee deemed further approval unnecessary, considering that that the main content of the manuscript is a review of the literature.

## RESULTS

**Figure 3:**
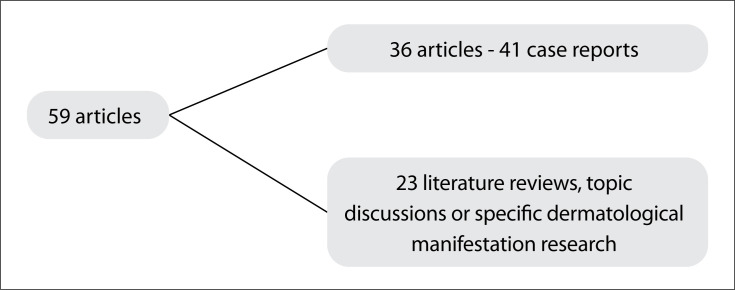
Article selection flowchart.

Fifty-nine of the 138 articles retrieved were selected, after applying the inclusion and exclusion criteria described above. Among these 59 articles, 23^13–34^ consisted of literature reviews, topic discussions or research focusing on specific dermatological manifestations, which were used to support the introduction and discussion of the present article (Figure 3). The remaining 36 articles^35–68^ referred to 41 case reports on patients with cutaneous manifestations secondary to nutritional deficiencies after bariatric surgery, whose data are compiled in Table 1. Five of them were papers presented at two congresses and were referenced together.^25,47^ The mean age was 44 years (range 29–66). The mean time between the day of surgery and the dermatological manifestation was 6.4 years (range 3–16).

**Table 1:** Summary of findings from the literature search, detailing the deficient mineral or vitamin, the associated dermatosis, type of procedure, age, gender, evolution and associated systemic involvement

Article (Ref.)	Nutritional deficiency after surgery	Dermatological manifestation	Type of surgery	Age	Sex	Adhered	Time to signs	Rx	Evolution	Systemic manifestations
Lopez et al.^52^	Zn	Pellagra-like erythema	VBG	51	F	DNA	5	O + A	Resolved in a few days	U
Bae-Harboe et al.^39^	Zn	Acrodermatitis enteropathica acquisita	RYGP	62	M	U	3	O	Resolved in a week	U
Mankaney et al.^53^	Zn	Acrodermatitis enteropathica acquisita	RYGP	54	F	A	8	U	Significant improvement	U
Rana et al.^59^	Zn, Vit. B6, Se, Alb	Acrodermatitis enteropathica acquisita, xerosis, diffuse non-scarring alopecia.	RYGP	39	F	A	13	P	Significant improvement	U
Jacob et al.^47^	Zn, Cu	Erythematous rash on the back and limbs	RYGP	54	M	U	10	P+O	U	Neurological, hematological.
Cunha et al.^41^	Zn, Cu, Fe, Alb	Acrodermatitis enteropathica acquisita, xerosis, pruritus	VBG/JIB	30	F	A	0.6	O	Significant improvement	Neurological, hematological
Stephens et al.^61^	Fe, Vit A + D	Photosensitivity, hair loss, discolored hair.	BPD	30	F	DNA	5	O	U	Endocrinological, neurological, ophthalmological
Boutin et al.^40^	Vit A+B6+B9+D, Alb	Generalized skin pigmentation	RYGP	44	F	DNA	4	O	Resolved	General, hematological
Messenger et al.^54^	Zn, Vit B3, Alb	Kwashiorkor, cracked eczema-like lesions	RYGP	35	F	U	10	P	Resolved	Hepatic steatosis
Panetta et al.^57^	Vit A	Acrodermatitis enteropathica acquisita, eczematous plaques on the limbs	BPD	51	M	U	.	P	Improved, but hyperpigmentation persisted	Ophthalmological
Vallabhaneni et al.^64^	Vit A + D	Phrynoderma	VBG/DS	50	F	U	NI	U	U	Polyarthralgia, fever
Wilson et al.^67^	Zn, Vit A + D	BADAS (bowel-associated dermatitis-arthritis syndrome). Ulcers and pustules on the limbs.	BPD	40	F	A	6	P+O	Died	Osteoporosis, ophthalmological
Zouridaki et al.^68^ Case 1	Vit B12 + D	Acrodermatitis enteropathica acquisita, pallor, hair loss, pruritic papular rash on back and limbs.	RYGP	45	F	U	2	P+O	Improved in 3 months	U
Zouridaki et al.^68^ Case 2	Vit B12	Leg ulcers, round scars on forearms, impetigo-like lesions on the scalp, localized alopecia, angular cheilitis	RYGP	62	F	DNA	8	P	Resolved in 6 months	Hematological
Gillette et al.^47^	Vit B1, B3, Alb, Fe	Large abscesses and ulcers on lower limbs	RYGP	47	F	U	10	BR+O	Improved in a year.	Neurological
Ashourian et al.^38^	Zn, Vit B3, B6	Kwashiorkor, thinning hair, xerosis on the trunk, hyperpigmented, macular rash	RYGP	32	F	U	0.3	O	Significant improvement in 4 weeks	None
Katugampola et al.^49^	Vit A, D, E, K	Acrodermatitis enteropathica acquisita, pellagra	JIB/GJ	66	F	DNA	12	BR	Resolved, but died after 7 months.	CKD, arthropathy, anemia
Vick et al.^66^	Zn, Cu	Erythema nodosum	RYGP	38	F	A	10	P	Significant improvement	U
Jaffe et al.^48^	Zn, Alb	Acrodermatitis enteropathica acquisita	GJ/SS	48	F	U	16	U	U	Gastrointestinal, malnutrition, coma
Garg et al.^46^ Case A	Zn, Vit A, Cu, Alb	Kwashiorkor, cheilitis, “cracked enamel” appearance on buttocks, thighs, and arms	RYGP	60	F	U	9	P	Died	Weakness, gastrointestinal.
Garg et al.^46^ Case B	Zn, Cu	Acrodermatitis enteropathica acquisita	RYGP	46	F	U	10	P	Significant improvement	Weakness, neurological, gastrointestinal.
Shackelton et al.^60^ Case 1	Zn, Cu, Vit A	Kwashiorkor, hair loss	RYGP	36	F	U	10	U	Resolved	U
Shackelton et al.^60^ Case 2	Zn	Acrodermatitis enteropathica acquisita	RYGP	45	F	U	0.7	U	Resolved, but intertrigo persisted.	U
Shackelton et al.^60^ Case 3	Zn, Cu, Vit A, B3	Acrodermatitis enteropathica acquisita	RYGP	32	F	U	3	U	Resolved	U
Garcovich et al.^45^	Zn, Fe, Vit D	Acrodermatitis enteropathica acquisita	BPD/DS	47	M	A	2	U	U	Endocrinological
Levenbergh et al.^51^	Vit B3, C, Alb	Hidradenitis suppurativa; phrynoderma	RYGP	58	F	U	1	O	Resolved in a few days	Gastrointestinal
Abad et al.^35^	Zn, Vit A, D3, E	Pellagra	BPB	46	M	A	13	O+P	Resolved in 10 months	Hematological
Ocon et al.^56^	Vit A, D, Fe	Phrynoderma	BPD	54	M	DNA	1	O+P	Resolved in 2 months but hyperpigmentation persisted	Osteoarticular, gastrointestinal, ophthalmological
Monshi et al.^55^ Case 1	Zn, Vit A, D, E, Alb	Phrynoderma	RYG	31	F	U	4	P	Improvement in 5 months	U
Monshi et al.^55^ Case 2	Zn, Vit A, B9, D	Phrynoderma; sharply demarcated perianal scaly plaques.	RYGP	29	F	U	7	P	Resolved in 7 days	U
Khanal et al.^50^	Zn	Acrodermatitis enteropathica acquisita	RYGP	45	F	A	0.1	P	U	Coma
Al Alawi et al.^36^	U	Facial and extremity macular rash	LOLGB	40	F	U	2	BR	U	U
Ramos-Levi et al.^58^	Zn, Vit A, D, E, Se, Cu, Alb	Hair loss, dry, pale skin.	RYGP/DS	48	F	A	2	BR	Improvement in 6 months	Ophthalmological, gastrointestinal
Velazquez et al.^65^	Fe	Xerosis; eczema and pruritus on back and limbs.	RYGP	34	F	A	0.3	O	Improvement in 3 months	Neurological
Vales-Montero et al.^63^	Vit A, B12, D	Hair loss	RYGP	40	M	DNA	5	P	Improvement in 3 months	Ophthalmological
Al-Douri et al.^37^	Zn, Cu	Papulosquamous lesions on limbs	RYGP	41	F	U	5	O	U	Gastrointestinal, osteoarticular, neurological
Evans et al.^42^	Zn, Cu	Hyperpigmentation of forehead, upper back, and upper arms	RYGP	40	F	U	NI	U	U	Steatohepatitis
Freitas et al.^43^	Zn, Vit A, B1, B6, C	Acrodermatitis enteropathica acquisita, pili torti, hair loss with short, brittle, lusterless hair.	RYGP	39	F	A	6	P	Significant improvement in 14 days	Neurological
Garcia et al.^44^	Zn, Vit B1, B6, C, Alb	Acrodermatitis enteropathica acquisita	RYGP	39	F	U	4	U	U	U
Sung et al.^62^	Zn	Acrodermatitis enteropathica acquisita. Eczema craquelé-like plaques.	RYGP	35	F	U	4	O	U	Hepatic
Wang et al.^25^	Zn	Palpable, painful purpuric rash on lower trunk and limbs. Desquamation of the dorsa of feet.	RYGP	37	F	U	5	P	Resolved	Gastrointestinal

Title lines: article (ref.): author's name and year of publication; Nutritional deficiency after surgery: nutritional deficiency status after bariatric surgery; Dermatological manifestation: describes skin manifestations; Type of surgery: type of bariatric surgery; Age: patient's age at the time of the report; Adhered: adherence to nutritional complementation; Time to signs: time (in years) between the date of the surgery and the dermatological manifestation; Rx: treatment offered; Evolution: Skin lesions follow-up and evolution; Systemic manifestations: systemic manifestation associated. Zn = zinc; Se = selenium; Cu = copper; Alb = albumin; Fe = iron; U = data unknown; VBG = vertical banded gastroplasty; RYGP = Roux-en-Y gastric bypass; VBG/JB = vertical band gastroplasty and jejunoileal bypass; VBG/DS = vertical band gastroplasty with duodenal switch; JIB/CJ = jejunoileal bypass and cholecysto-jejunostomy; GJ/SS = gastrojejunostomy and stomach stapling; BPD/DS = biliopancreatic diversion with duodenal switch; BPB = biliopancreatic bypass; LOLGB = gastric bypass with laparoscopic omega loop; RYGP/DS = RYGP and duodenal switch; Adhered: to the supplementation protocol. DNA: did not adhere; O+A: oral supplementation of nutrients and antibiotics; O: oral nutrient supplementation; P: parenteral nutrient supplementation; P+O = parenteral and oral nutrient supplementation; BR = bariatric reversal; BR+O = bariatric reversal and oral nutrient supplementation; BR+P = bariatric reversal and parenteral nutrient supplementation.

The most frequent type of bariatric surgery among the patients who developed dermatological manifestations was Roux-en-Y gastric bypass (70%), followed by biliopancreatic bypass (14%). Among the articles that reported whether the patient adhered to post-surgical nutritional supplementation (18/41 patients; 44%), we found that only 61% of them adhered to this. Most of the patients (75%) had multiple-need malnutrition and the deficiencies identified were the following: zinc (68%); vitamin A (36%); vitamin D (34%); albumin (29%); copper (27%); vitamins B3 and B6 (12% each); and iron, ferritin, folic acid, vitamins B1, B12, C and E and selenium (1% or less each). The most common manifestation, found in 39%, was acquired acrodermatitis enteropathica, which was significantly associated with bariatric surgery consisting of Roux-en-Y gastric bypass (P value < 0.001), and which bore no relationship with the post-surgical time interval. This manifestation was followed, in frequency, by xerosis (31%), hair loss (22%) and pellagra-like lesions (12%).

## DISCUSSION

After bariatric surgery, regular multidisciplinary follow-up of the patients must be maintained for many years. It includes adoption of a balanced diet, supplementation with minerals and vita-mins and periodic laboratory evaluations. However, despite the recommendations, studies^69^ have shown that these patients are often monitored irregularly and that their adherence to vitamin and mineral supplementation is low.

Our search in the literature showed that only 61% of the patients who developed dermatological signs of nutritional deficiencies adhered to supplementation. Sunil et al.^70^ found that being female, not having a full-time job and having symptoms of attachment anxiety were factors that related to lower adherence to post-surgical recommendations. Mahawar et al.^71^ reported that the main reasons given by patients for non-adherence to supplementation were: 1 – difficulty remembering to take the pills; 2 – high number of pills; 3 – the advent of side effects; and 4 – financial cost. In addition, it needs to be considered that patients can present nutritional deficiencies even before being treated through bariatric surgery and that these conditions are worsened in the postoperative period, over both the short and the long term.^72^

This review showed that most nutritional deficiencies present with dermatological manifestations at a relatively early stage (53% within five years or less after the date of surgery). The most common manifestation was acquired acrodermatitis enteropathica.^73^ This is associated with zinc (Zn) deficiency and presents with eczematous plaques over the extremities and around natural orifices. Zn and Zn transporters have many physiological functions in the skin: Zn is a cofactor of hundreds of enzymatic reactions, is an important structural component of gene regulatory proteins, regulates apoptosis and is fundamental for protein synthesis and RNA packaging. Zn binds to approximately 10% of human proteins. Therefore, this metal is associated with many organic activities, such as cell development, differentiation and growth, and Zn deficiency manifests itself in several ways, in all organs.^74^ It should be noted that zinc supplementation may impair copper absorption, and in bariatric surgery patients with low zinc levels it is recommended that oral copper should be administered separately.^75^

The second most-common manifestation was pellagra-like lesions, which manifest as symmetrical erythematous plaques that can be accompanied by edema and sometimes blisters, located on sun-exposed areas of the skin.^76^ Pellagra is caused by a deficiency of niacin also known as nicotinic acid, a form of vitamin B3. Niacin is absorbed from the diet, or synthesized from the amino acid tryptophan, and is important for synthesis of NAD and NADP: coenzymes that generate high-energy phosphate bonds that are essential for the metabolism of glucose, amino acids and proteins. Niacin deficiency will be felt first in tissues with a high cell turnover, such as the intestines (manifesting as diarrhea) and the skin, and in organs with high energy demands, such as the brain (causing insomnia, fatigue, nervousness, irritability and depression).^77^

The next most-common complaints were xerosis and hair thinning, which have been correlated with deficiencies of various nutrients, particularly fat-soluble vitamins, given that malabsorptive surgery impairs the absorption of lipids.^78^ Retinoic acid (vita-min A) is a biologically active retinoid with life-sustaining functions, and severe deficiency of this can result in illness and death. Retinoids fulfill many essential physiological processes and are critical for normal growth, development of normal skin and eyesight, an effective immune system and fertility. Normal levels of this vitamin depend on consumption, and its metabolism is complex, mediated by triglycerides and cholesterol.^79^ Vitamin A deficiency can affect the eyes, causing night blindness and xerophthalmia,^80^ and can also cause skin changes, such as phrynoderma and perforating folliculitis with elimination of elastic fibers. This can lead to considerable scarring, as occurred in the example above.^81,82^

Regarding vitamin D3, epidermal keratinocytes are a primary source of vitamin D for the body, as they have the enzymatic machinery to metabolize it into its active metabolite 1α,25-dihydroxyvitamin D. They also express the vitamin D receptor and can thus respond to the vitamin D3 that they produce. This vitamin has essential functions in the skin, including control of cell proliferation and differentiation and stimulation of innate immunity and the hair cycle. The actions of vitamin D on the skin are controlled by two classes of coactivators: vitamin D receptor interacting proteins (DRIPs) and the p160 steroid receptor (SRC) family of coactivators.^83^

Another important element for skin health is vitamin E, which is not synthesized by humans and must be consumed in the diet. There are eight types of vitamin E, and γ-tocopherol is the most abundant type found in foods, while α-tocopherol is the most abundant type found in human tissues and serum. In human skin, vitamin E inhibits production of prostaglandin E2 and nitric oxide and prevents oxidative stress from ultraviolet (UV) radiation, manifested through formation of sunburn cells, lipid peroxidation and edema. Vitamin E has a protective role against cancer because it reduces formation of UV-induced photo adducts and immunosuppression.^84^

Vitamin B12 deficiency was also commonly detected (Table 1) and presented in the forms of angular cheilitis, glossitis, hair depigmentation and mucocutaneous hyperpigmentation. Methylcobalamin and adenosylcobalamin are two biologically active forms of vita-min B12. Methylcobalamin is a coenzyme with methionine synthase that is essential for synthesis of pyrimidines and purines, while adenosylcobalamin degrades fatty acids. Excess or deficiency of cobalamin can lead to dermatological manifestations, due to changes in the complex biochemistry and metabolism of this vita-min.^85^ Excess B12 can produce acneiform reactions, as it modulates the transcriptional activities of *Propionibacterium acnes*. This is an indication that development of the disease depends on the interaction mediated by metabolites between the host and the skin flora.^86^ If untreated, patients with this deficiency may present with megaloblastic anemia and even neurological manifestations such as paresthesia, loss of positional awareness and cognitive impairment.^77,87^

As demonstrated in Table 1, among the 41 patients selected, 27 had systemic manifestations and these were potentially fatal or fatal in 11 patients.^37,41,43,46-47,49,50,61,65,67^

Although not the purpose of this review, it is also necessary to consider that there are cutaneous manifestations that occur after bariatric surgery, which are not due to nutritional deficiency. On the contrary, these are due to excessive replacement of micronutrients, a situation seen with relative frequency in patients in the late postoperative period of bariatric surgery.^86^

Discussion of the clinical guidelines for nutrition and metabolic support for patients under follow-up after bariatric surgery was beyond the scope of the present study.^88^

Ha et al.^89^ published a systematic review and meta-analysis of longitudinal studies on bariatric surgery patients who received postoperative supplementation in accordance with the guidelines. They found that despite adequate monitoring and treatment, there were significant reductions in micronutrient levels at 11 postoperative time points, with a moderate level of evidence (vitamin A at 12–23 months, vitamin E at ≥ 24 months after Roux-en-Y gastric bypass and ferritin at ≥ 24 months after sleeve gastrectomy). Their results help in understanding the optimal micronutrient monitoring times for these patients. They stated limitations to their study, which were identical to ours: few studies included detailed information on patients’ adherence to the recommended treatment, and most articles did not describe the dietary modifications after surgery. The latter is an important point to be considered, as micro-nutrient absorption is affected by food intake.

In our series, none of the articles described the prescribed supplementation regimens and there was no information on the pre-operative nutritional status of the patients. Further studies that are prospective and well-controlled should be conducted to generate more accurate data, so that the consequences of bariatric surgery on the skin can be properly evaluated.

## CONCLUSION

Patients who underwent bariatric surgery must be monitored frequently for mineral and vitamin deficiencies. Skin diseases can be a manifestation of such conditions and should prompt immediate investigation of the patient's nutritional status.
